# Filling the Network Gap in Research Ethics: Analyzing Ethical Issues at Scale in Big Team Science

**DOI:** 10.1002/hast.70046

**Published:** 2026-07-20

**Authors:** Susan M. Wolf, Gillian H. Roehrig, Timothy L. Pruett, Korkut Uygun, Adam Koch, Claire Colby McVan, Evelyn Brister, Shawneequa L. Callier, Alexander M. Capron, James F. Childress, Rosario Isasi, Andrew D. Maynard, Kenneth A. Oye, Paul B. Thompson, Terrence R. Tiersch

**Keywords:** research ethics, research network, network ethics, responsible conduct of research, team science, engineering ethics

## Abstract

*Scientific research increasingly involves large, multidisciplinary teams networked across multiple institutions to develop new technologies. Despite the rise of complex research networks and big team science, there has been too little analysis to date of the ethical challenges facing these networks. The extensive literature on the ethical issues confronting individual researchers and small teams (the microlevel) and on the larger societal challenges flowing from research and new technology (the macrolevel) leaves a troubling gap in between, at the mesolevel of the research network involved in big team science. Yet the ability of complex networks to conduct research ethically—which is essential if the results are to be deemed reliable and trustworthy—depends on recognizing the ethical issues that emerge at this intermediate network level, identifying the values that should guide networks in addressing those issues, and equipping research leaders to build a culture supporting the ethical conduct of research across the laboratories and institutions that comprise the network. This paper calls out the problem, analyzing the gap and recommending next steps*.

## Article

In the past several decades, scientific research has evolved. Research has been increasingly conducted by large investigator groups spread over multiple institutions, disciplines, and locations.^1^ This transformation has fueled the growth of the science of team science (SciTS) as a field of study that studies “how teams connect and collaborate to achieve scientific breakthroughs that would not be attainable by either individual or simply additive efforts.”^2^

The rise of team science has also drawn the attention of the National Academies of Sciences, Engineering, and Medicine. In 2015, the National Research Council issued a landmark report on the effectiveness of team science, defining larger teams as those composed of more than ten scientists while noting that some include hundreds or more.^3^ In 2025, the National Academies published a follow‐up report, *The Science and Practice of Team Science*.^4^

Yet, to date, the team science literature has offered little analysis of the ethical issues that arise in conducting big team science. Certain topics—such as the greater complexity of authorship disputes that may erupt in large scientific teams^5^ and the threat these disputes may pose to successful collaboration^6^—have received attention, but analysis of the range of ethical issues arising at the level of a research network remains underdeveloped.

This gap is a problem. The increasing scale and complexity of scientific research projects—including what the organizational and SciTS literatures call “teams of teams” or “multiteam systems”^7^—raise ethical challenges that beg for analysis. Among these are questions of when and how such a research network should harmonize norms and conduct across laboratories, institutions, disciplines, and jurisdictions; how research leaders should manage these complex networks to foster responsible research, deter misconduct, and discharge the proliferating duties imposed by funders, oversight authorities, and regulators; and what steps research networks spread over multiple institutions should take to earn public trust and confidence.

Failure to address such issues can lead to serious problems—cross‐laboratory collaborations thwarted by conflicting approaches to data sharing and authorship, research misconduct undetected by distant collaborators, trainees facing conflicting demands in multilaboratory collaborations, community partners and research participants mystified by inconsistent messaging and approaches across the network, and decisions by network leaders that undermine rather than advance the responsible conduct of research (RCR). For example, pursuit of the network's scientific goals may require researchers based in a laboratory at one institution to collaborate with those in another laboratory and institution, only to discover later that the two laboratories have fundamentally different approaches to determining authorship credit, author order, and even where to publish. The principal investigators directing those laboratories may not be able to resolve the dispute, especially if each is committed to the soundness of their own approach. If the leaders of the overarching network do not anticipate such issues and establish network‐wide norms to encourage early negotiation of authorship issues and to limit the range of authorship approaches in collaborating laboratories to those ethically appropriate for the network, such disputes are likely to recur, eroding trust, undermining commitment to ethical authorship practices, and thwarting collaboration.

Other examples abound. Sloppy or even fraudulent research practices may go undetected by remote collaborators as the network stretches across institutions whose commitment to research integrity and oversight programs varies. Unless network leaders build a culture of research ethics and integrity, create network‐wide systems of transparency and accountability (such as data‐sharing mechanisms), and actively address emerging issues, the network risks serious ethical, scientific, and reputational consequences.

These are only some of the problems that can arise when network leaders neglect the need to address research ethics at the network level. Analysts of large and dispersed scientific teams have pointed to others, including risks posed by weakened supervision for trainees across distances, researchers unaware that some members of a big team may have conflicts of interest, and the challenges of handling misconduct allegations when teams cross multiple institutions.^8^ If network leaders do not anticipate these risks, creating norms and practices to manage them—such as network guidance on the range of ethical and acceptable approaches to authorship, data sharing, and mentoring—then the network's ability to achieve its scientific goals by conducting ethical and trustworthy research may be compromised.

Thus far, the field of research ethics has largely failed to address these heightened risks in big, dispersed research networks. For decades, work on research ethics has focused mainly on *microethics*—the duties of individual researchers, laboratories, or small teams. Research ethics sometimes also tackles the *macroethics* of large‐scale societal implications.^9^ What requires more attention are the *mesoethics* issues in the middle—challenges that can arise in scientific research networks designed to cross multiple institutions and disciplines. (See table 1.)

**Table 1:** Three Levels of Analysis for Research Ethics in Science

*Ethics level*	*Primary focus*	*Examples of ethical issues*	*Adequacy of literature & tools*
Micro	Individual investigators or small teams working in a single laboratory	Avoiding plagiarism and other research misconduct, disclosing and managing conflicts of interest, minimizing detrimental research practices	Extensive literature and training tools to support the ethical and responsible conduct of research (RCR)
Meso	Multilaboratory, multi‐institutional research networks	Assessing differences across a network in approaches to issues such as authorship and data sharing, promoting cross‐network transparency and collaboration, creating processes to support a network‐wide approach to ethics and RCR issues	Limited literature on key network values; need for tools to identify ethical issues in networks and for training materials to equip network leaders to promote ethical and responsible network conduct
Macro	Large‐scale societal impacts	Minimizing risks while maximizing benefits to society, consulting those groups potentially affected by the scientific research and resulting innovations	Significant literature offering methods such as risk analysis, horizon forecasting, anticipatory governance, and responsible innovation

Failure to address the ethical issues arising at the mesolevel of a research network leaves an important gap. In comparison, attempting to understand a corporation by studying only the microbehavior of individuals and small teams or the macroimpact on society would be seen as obviously inadequate; social network analysis and organizational theory are now too well established to omit analysis at the mesolevel.^10^ Research ethics needs to meet this challenge with both empirical study of the ethical issues that emerge in large‐scale research networks and normative analysis of how to conceptualize and address the mesolevel problems in research ethics.

This paper is a call to fill that gap by developing research ethics at the mesolevel of the research network, defined here as a collaborative research structure formally created to fulfill overarching scientific goals by crossing institutions, laboratories, and (often) disciplines.^11^ Developing research ethics at the mesolevel will complement, not replace, existing research ethics at the micro‐ and macrolevels. Note that, while some research networks span countries with different norms and rules on research ethics, leading to cross‐border challenges, we focus here on U.S. research collaborations to begin the needed work of developing network ethics.

## The Challenge of NetEthics

Funded by the National Science Foundation, our NetEthics project group set out to map this mesoterrain using mixed methods. We analyzed the literature on research ethics, big team science, and organizational theory. At the same time, we studied in detail a well‐established model of network research by focusing on a designated Engineering Research Center (ERC) funded by NSF starting in 2020.^12^ ERCs exemplify big team science spread over multiple institutions, disciplines, and locations.^13^ Each ERC pursues “a deeply collaborative, team‐based engineering approach for defining and solving important and complex societal problems.”^14^ Indeed, the National Academies have called ERCs the NSF's “flagship program” for “center‐scale research.”^15^ The ERC funded to pioneer Advanced Technologies for the Preservation of Biological Systems (ATP‐Bio) became the petri dish for our study of network ethics.^16^

This project's analysis of network ethics has required attention to terms and concepts rarely used in work on research ethics. Table 2 lists terms commonly used to refer to scientific teams and their research: “team science,” “multiteam systems,” “team of teams,” “research network,” and “big team science.”

**Table 2:** Terms Used in the Literature to Refer to Scientific Teams and Their Research

**Team science:** “Scientific collaboration, i.e., research conducted by more than one individual in an interdependent fashion, including research conducted by small teams and larger groups.”^1^
**Multiteam system:** “[T]wo or more teams that interface directly and interdependently in response to environmental contingencies toward the accomplishment of collective goals. MTS boundaries are defined by virtue of the fact that all teams within the system, while pursuing different proximal goals, share at least one common distal goal; and in doing so exhibit input, process, and outcome interdependence with at least one other team in the system.”^2^
**Team of teams:** “[A] network of coordinated teams who are interdependent and share trust as well as an awareness of a common purpose, and goal.”^3^
**Research network:** “Research networks are a specific type of networked organization that are, in essence, networks of teams or work units progressing towards a common goal.”^4^ “Institutional research networks refer to the formal organizations, funded through public or private sources for a set period to advance specific goals, such as strengthening research capacity.”^5^
**Big team science:** “A method involving a relatively large number of collaborators who may be dispersed across labs, institutions, disciplines, cultures, and continents.”^6^ Those larger groups have been defined to include more than ten individuals who conduct team research, and sometimes many more.^7^ “Such very large groups typically possess a differentiated division of labor and an integrated structure to coordinate the smaller science teams.”^8^

1. National Research Council, *Enhancing the Effectiveness of Team Science* (Washington, DC: National Academies Press, 2015), 22. The National Academies 2025 report on team science similarly defines this term as “collaborative, interdependent research conducted by more than one individual.” National Academies of Sciences, Engineering, and Medicine, *The Science and Practice of Team Science* (Washington, DC: National Academies Press, 2025), 11, 203, 246.

2. J. E. Mathieu, M. A. Marks, and S. J. Zaccaro, “Multiteam Systems,” in *Handbook of Industrial, Work and Organizational Psychology,* vol. 2, *Organizational Psychology*, ed. N. Anderson et al. (London: Sage, 2001), 290, doi:10.4135/9781848608368.n16. “As an organizational form, the multiteam system structure is becoming increasingly prevalent, particularly in scientific endeavors, as it allows for exploring and investigating more complex challenges.” National Academies of Sciences, Engineering, and Medicine, *The Science and Practice of Team Science*, 214.

3. E. M. Raybourn, J. D. Moulton, and A. Hungerford, “Scaling Productivity and Innovation on the Path to Exascale with a ‘Team of Teams’ Approach,” in *HCI in Business, Government and Organizations: Information Systems and Analytics*, ed. F. Fui‐Hoon Nah and K. Siau (Cham, Switzerland: Springer, 2019), 408‐21, at 413, citing S. McCrystal et al., *Team of Teams: New Rules of Engagement for a Complex World* (New York: Penguin Random House, 2015).

4. G. Y. Mo, Z. Hayat, and B. Wellman, “How Far Can Scholarly Networks Go? Examining the Relationships between Distance, Disciplines, Motivations, and Clusters,” *Communication and Information Technologies Annual* 9 (2015): 107‐33, at 108.

5. D. Contandriopoulos, C. Larouche, and A. Duhoux, “Evaluating Academic Research Networks,” *Canadian Journal of Program Evaluation* 33, no. 1 (2018): 69‐89, at 71.

6. P. S. Forscher et al., “The Benefits, Barriers, and Risks of Big‐Team Science,” *Perspectives on Psychological Science* 18, no. 3 (2022): 607‐23, at 608.

7. National Research Council, *Enhancing the Effectiveness of Team Science*, 2.

8. Ibid.

In the literature on SciTS, we found approaches seldom deployed in bioethics. Analyses of the competencies necessary for success in big team science emphasize the trajectory of collaboration as teams form and consolidate, values such as psychological safety, the ability to navigate conflict, and the necessity of shared goals and effective network leadership. While research ethics and bioethics more generally have emphasized foundational principles, reasoning from key cases, and core virtues, the literature on big team science has focused on the realities of human and team behavior and the real‐world challenges of dispersed teams comprised of researchers from multiple disciplines collaboratively striving to solve demanding scientific problems.^17^ And while research ethics is typically anchored in prescriptive documents such as the Nuremberg Code and Declaration of Helsinki as well as the regulatory language of the Federal Policy for the Protection of Human Subjects (the Common Rule) and the U.S. Food and Drug Administration's equivalent, as interpreted by institutional review boards (IRBs) and other oversight authorities,^18^ the team science literature commonly references social psychology, insights from organizational and management theory, and studies of creativity and conflict.

Successfully expanding research ethics to address scientific work by complex, multi‐institutional research networks thus requires development on two fronts—normatively evolving research ethics to address the choices and obligations arising at the mesolevel of the formal research network and empirically enlarging the scope of research ethics work to address the behavior and functioning of these large and complex research organizations. We address both in turn.

## Evolving the Norms of Research Ethics to Address Networks

A focus on the ethical obligations of individual investigators—the microlevel—is clear from the start of modern research ethics. Twentieth‐century research ethics counts the Nuremberg Code, the revelation of the Syphilis Study at Tuskegee, and later *The Belmont Report* ^19^ as milestones, leading to the federal regulations on human subjects research issued by the Department of Health and Human Services^20^ and the FDA.^21^ Yet the ethical duties articulated were typically duties falling on individual researchers or small teams. Even when scholars recognized that research atrocities were deeply influenced by group phenomena such as the Nazi war effort, pervasive racism in the Syphilis Study at Tuskegee, and widespread failure to recognize the profound vulnerability of institutionalized individuals at Willowbrook State School, research ethics largely became the responsibility of individual researchers, overseen by their individual research institutions. Indeed, the Nuremberg court properly held individuals accountable for the harms they inflicted, deliberately rejecting the defense of superior orders (the Nuremberg defense) that would have exonerated the individual because they were following orders as part of a larger organization.

Of course, the locus of ethical responsibility is not an either‐or matter—responsibility can fall both on individuals and on the larger system and its leaders. Yet network ethics has remained undeveloped. The rise of modern research ethics beginning after World War II and later the exposure of the Syphilis Study at Tuskegee led to regulations establishing research oversight bodies such as IRBs. These oversight bodies recognized organizational responsibility, but largely within single research institutions. It is no surprise that the National Academies’ most popular publication, *On Being a Scientist* (first published in 2009 and now in its third edition), was addressed to individual investigators.^22^ Only recently have the Academies considered whether an additional publication is needed addressing the question of how to lead larger research teams, organizations, and networks to ensure ethical and responsible research.^23^

To be fair, bioethics has considered organizational issues in some contexts, especially in health care delivery and public health. In health care, a literature on organizational ethics has recognized that hospitals and other organizations have obligations to patients and their communities. But the focus has typically been on the ethical responsibilities of single organizations as well as their processes for addressing ethical issues in the care of individual patients.^24^ In the Covid‐19 pandemic, public health concerns impelled many health care institutions to articulate policy for allocating treatment interventions across patients under conditions of acute scarcity and to consider when to declare crisis standards of care that would prioritize population health over the traditional commitment to individual patient welfare. However, studies now reveal that crisis standards were rarely declared, institutions typically defaulted to conventional standards for dyadic doctor‐patient care even when scarcity called for a shift to population‐based standards, and coordination across institutions often failed.^25^

In the field of research ethics, attempts to address large and complex research networks have been limited. When the U.S. government revised the Common Rule in 2017, the published announcement of the final revision included a prefatory explanation acknowledging “a paradigm shift … in how research is conducted,” with changes including the emergence of “[c]linical research networks connected through electronic health records.”^26^ Yet, as the announcement recognized, “the human subjects research oversight system … has remained largely unaltered over the past two decades.”^27^

The Common Rule revision offered an opportunity to evolve the rules for research with human participants to address the reality of big team science in formal research networks crossing institutions. However, the portion of the actual revision addressing this fundamental change focused on “a requirement for U.S.‐based institutions engaged in cooperative research to use a single IRB for that portion of the research that takes place within the United States.”^28^ Even this limited response met with substantial opposition,^29^ despite a long history of efforts—including policy for NIH‐funded studies^30^—to encourage multisite studies to use a single IRB (sIRB).^31^ As the revision announcement explained, “Arguments against the proposal cited the need for local review and potential loss of accountability, as well as operational issues such as the increased administrative capacity and technological systems required for a site to function effectively as a single IRB.”^32^ Despite the opposition, the sIRB requirement was incorporated in the Common Rule revision.^33^ This effectively required the creation of multi‐institutional research networks, at least for the purposes of IRB oversight of multisite studies. Yet implementation has proven challenging,^34^ and “the impact of sIRB review on protections for human research subjects and the ethical integrity of research remains unclear.”^35^

The embattled sIRB requirement in the Common Rule revision was an anemic response to the fundamental transformation in the conduct of research suggested in the revision's published announcement. As research groups have grown in size and complexity, work on research ethics has not yet addressed the challenges emerging at the network level. Faced with the realities of large‐team science, the temptation has been to stick with the individual as the unit of analysis, continuing to ask what individual participants in a team should do, without adequate attention to how research networks should identify and respond to ethical issues. Confronting the ethical challenges that arise in formal networks that are often geographically dispersed, multidisciplinary, and characterized by a complicated web of connections (including cross‐cutting groups defined by collaborative projects) requires more. Individual ethics and accountability remain essential, but so is attention to the network structures, processes, culture and climate, and mission needed to support the ethical and responsible conduct of research by the network itself.

Thus, the unit of analysis at the mesolevel of research ethics needs to be the network itself. A formal research network is composed of individual actors in different roles, but it is also characterized by structures, policies and processes, culture and climate, and mission. As noted above, without guidance and a common approach at the mesolevel, a research network may face widely divergent approaches across component laboratories to core issues, including the importance of adhering to ethical norms in research with animals and human participants.^36^ Network scientists collaborating on projects that cross laboratories and research institutions may not know whether they can trust the work of their collaborators. Without a common approach and effective network leadership, misunderstandings, disputes, and even scientific error may arise. Indeed, the 2015 National Research Council analysis of team science found that “what is important for successful collaboration changes dramatically as the number of participants grows.”^37^ That report identified seven team features that can produce challenges: member diversity, cross‐disciplinarity, team size, goal misalignment across labs, changing membership, being dispersed geographically, and conflict arising from task interdependence.^38^

The National Academies 2017 report *Fostering Integrity in Research* similarly identified risks of collaborative science, such as “miscommunication, misunderstandings, unrealistic expectations, and unresolved disputes.”^39^ Other work considers cross‐disciplinary teams whose members may have experienced different ethics and RCR training, which suggests the need for a more unified approach.^40^ Effective leadership is crucial to establishing a culture of ethics and to modeling responsible conduct of research on scientific teams.^41^

Evolving the norms of research ethics to address the ethical issues that emerge in research networks will require a recognition that these mesolevel issues can take three basic forms: differences across investigators, laboratories, or institutions within the network that create barriers to network‐wide collaboration; issues facing the network itself to ensure the ethical and responsible conduct of research across the network and successful functioning as a network; and ensuring the network capacity to identify and address societal issues in developing the technologies under study. Table 3 delineates these three conceptual categories.

**Table 3:** Three Categories of Network Issues

**Differences at the microlevel that raise challenges for cross‐network collaboration** and functioning (such as problematic differences across network laboratories in their approaches to authorship or data sharing), requiring attention from network leaders rather than relying on lab‐specific or institution‐specific solutions.
**Issues facing the network itself that can be handled only at the network** leadership level (such as how the network should be structured and function, with what overarching goals, policies, and partnerships), also called **“purely meso”** issues.
**Ensuring that the network builds the capacity to anticipate and address societal challenges at the macrolevel**, including how to secure net societal benefit from the innovations.

In the first category—microlevel divergence that causes mesolevel problems—differences across laboratories, research groups, and institutions within the research network can create obstacles to cross‐network collaboration, requiring intervention from network leaders to support network progress. For example, ethically allocating credit across a large team can be problematic, as can resolving disputes about ownership of intellectual property and potential spin‐off companies.^42^ In addition, collaborating across disciplinary and cultural differences can surface conflicts when expectations, norms, and beliefs clash.^43^ Coordinating oversight bodies such as IRBs across different institutions that are collaborating to form a network can also be challenging and lead to conflicting results.^44^

In the second category—mesolevel issues facing the network itself—network leaders and actors will need to create a shared mission, vision, and set of goals across a large and dispersed multiteam system.^45^ Establishing consistent best practices for ensuring reproducible science is more difficult with a large number of geographically dispersed team members.^46^ Data management across a large team, including compliance with ethical and regulatory requirements for data privacy, can be challenging.^47^ Determining what stakeholders and interested parties the network should engage with and what entities the network should collaborate with in research, development, and technology translation are all mesolevel issues that can raise ethical issues, especially as large‐scale network science attracts commercial interest and prompts potential societal concerns. Dealing effectively with such issues requires understanding what network structures and processes are needed.

Finally, the third category requires network leaders to consider what personnel and processes are needed to ensure network capacity to address societal issues at the macrolevel. NSF, for example, has become increasingly explicit that ERCs are expected to build the capacity for upstream stakeholder engagement to codevelop emerging technologies with key publics and interested parties.^48^ Building this kind of capacity requires that the network include personnel with expertise in methods such as ELSI analysis (focusing on ethical, legal and societal implications), public engagement, and community‐based participatory research as well as other methods that allow early identification of key issues and that work to address them.^49^

These three categories of network issues suggest a number of key tasks for network leaders to address in forming and sustaining the network over time. However, further conceptual work is needed to identify all of the ethical and RCR issues facing networks and the relevant values they can use to address them. This will require analysis informed by insights from organizational theory and SciTS.

## Expanding Empirical Work on Research Ethics to Address Network Competencies and Functioning

Evolving research ethics to support the ethical functioning of research networks will also require empirical work informed by the literature on SciTS. That field developed to meet “the need for evidence‐based solutions to advance” team science.^50^ Much of the SciTS literature analyzes inputs, processes, and outputs in team science,^51^ where inputs include team composition and design, processes include how the team functions, and outputs include indicators of effectiveness.^52^

The SciTS literature emphasizes the competencies needed at multiple scales to succeed in team science, including individual capacities to collaborate, cross‐laboratory and cross‐institutional commitment to collaboration, and the leadership needed at the network level to build and sustain successful functioning over time.^53^ Articulating the specific competencies needed to ensure *ethical* functioning of the network will be essential, as will empirical study of the real‐world challenges and of what strategies work to meet those challenges. This means enlarging the universe of research ethics to integrate the tools of social psychology, organizational theory, and network analysis to address ethics at the scale of a multi‐institutional network.

Although the SciTS literature has much to contribute, the metrics used in that field to judge team effectiveness typically focus on achievement of scientific goals, number of publications, and other indicators of scientific impact—not indicators of ethical conduct, processes, and structures. This gap is an open invitation for those studying research ethics to contribute a much‐needed perspective to the study of big team science and research networks. What competencies are needed to ensure that a network performs research ethically? Managing a network to ensure ethical conduct may demand more than the knowledge and capacity to build teams, establish a shared vision, and organize scientific work to achieve project goals. It may require building guidance, training, processes, and oversight to address ethical responsibilities, support ethical conduct, identify problematic practices and divergence from network norms, and respond effectively. Network leaders need training in the ethical conduct of research at multiple scales, including at the level of the individual investigator, laboratory and small team, individual institution, and cross‐institutional network.

Understanding the ethical challenges that can erupt in complex research networks will thus require both empirical research and theoretical development. Our NetEthics project has combined the two, using semistructured interviews to study how participants in one scientific and engineering network perceive ethical issues.^54^ We have also developed a survey tool that network leaders can adapt to ascertain divergence and convergence across the network on ethically sensitive issues such as authorship credit and data sharing with other investigators prior to publication. Finally, we have developed case studies to catalyze group discussion and interactive learning within a network on key ethical issues.^55^

More needs to be done, studying network ethics across different kinds of research networks and investigating the effectiveness of different interventions.^56^ Making progress will also require assessing the effectiveness of guidelines, best‐practices documents, collaboration agreements, and other written tools designed to support ethical conduct across a multi‐institutional research network.^57^ Much of the literature on team science recommends the use of governing documents in collaborative research.^58^ NSF‐funded ERCs are structured by multiple documents, including those that each ERC must submit within ninety days of receiving an award.^59^ While some of these documents touch on issues relevant to network ethics (such as data management and documentation of IRB approval), none focus directly on how network leaders should ensure ethical and responsible conduct throughout the network.

## Developing Research Ethics at a New Scale

The rise of big team science has seen the growth of formal research networks crossing multiple research laboratories, institutions, and disciplines. Often, these networks are dispersed geographically and across time zones. NSF‐funded ERCs are only one example of a wide array of research networks, but ERCs exemplify the challenges these networks face in ensuring the ethical and responsible conduct of research.

Historically, scholarship about research ethics has neglected the mesolevel ethical issues associated with research networks. This paper starts to fill that gap by conceptualizing the types of issues that need attention and calling for empirical study of how actual networks address research ethics. Progress will require studying a wide range of research networks.

Developing research ethics at the mesolevel is essential to guide these networks. Accomplishing scientific goals requires ethical and responsible conduct. Without that, scientific results will not be deemed reliable and worthy of peer and public trust. Moreover, funders such as NSF and NIH that disperse public funds to support research networks require that these funded groups are accountable to the public. And research networks that are developing emerging technologies with the potential to produce tremendous benefit as well as profound disruption have a duty to pursue their innovative research and technology development responsibly.

Discharging these obligations requires long‐overdue attention to the mesolevel ethics of a research network. This calls for the development of research ethics at a new scale—that of a complex research network spanning laboratories, institutions, and disciplines, with investigators dispersed over time zones and locations. This new work will offer a much‐needed complement to the existing analyses of research ethics at the micro‐ and macrolevels of research. Supporting the ethical and responsible conduct of research in complex multiteam systems is the next frontier for research ethics.

## Acknowledgments and Disclaimer

Preparation of this paper was supported by National Science Foundation award 2220611 for the authors’ project, NetEthics: Building Tools & Training to Advance Responsible Conduct in Complex Research Networks Pioneering Novel Technologies. Any opinions, findings, and conclusions or recommendations expressed in this material are those of the authors and do not necessarily reflect the views of the National Science Foundation. Thanks to NetEthics project members, including Keisha Varma, Barbara J. Evans, Michele B. Goodwin, Insoo Hyun, and Gary E. Marchant, for input throughout the NetEthics project. Thanks also to Caleb Campbell, Ethan Wold, and Lowell Wolfe for research assistance; to Dori Henderson and the staff of the University of Minnesota's Consortium on Law and Values in Health, Environment & the Life Sciences for administrative support; and to the leadership and staff of the NSF Engineering Research Center for Advanced Technologies for the Preservation of Biological Systems (ATP‐Bio) (funded by award EEC 1941543) for support and input. This paper benefited from the lead author's membership in the Strategic Council for Research Excellence, Integrity, and Trust of the National Academies of Sciences, Engineering, and Medicine and service on the planning committee for the National Academies Workshop entitled “On Leading a Lab.”

## A Note about Authorship

All authors after the first six are listed in alphabetical order.
